# Improved reference genome for the domestic horse increases assembly contiguity and composition

**DOI:** 10.1038/s42003-018-0199-z

**Published:** 2018-11-16

**Authors:** Theodore S. Kalbfleisch, Edward S. Rice, Michael S. DePriest, Brian P. Walenz, Matthew S. Hestand, Joris R. Vermeesch, Brendan L. O′Connell, Ian T. Fiddes, Alisa O. Vershinina, Nedda F. Saremi, Jessica L. Petersen, Carrie J. Finno, Rebecca R. Bellone, Molly E. McCue, Samantha A. Brooks, Ernest Bailey, Ludovic Orlando, Richard E. Green, Donald C. Miller, Douglas F. Antczak, James N. MacLeod

**Affiliations:** 1 Department of Biochemistry and Molecular Genetics, School of Medicine, University of Louisville, Louisville, KY 40292 USA; 2 Department of Biomolecular Engineering, UC Santa Cruz, Santa Cruz, CA 95064 USA; 3 Genome Informatics Section, Computational and Statistical Genomics Branch, National Human Genome Research Institute, National Institutes of Health, Bethesda, MD 20892 USA; 4 Center for Human Genetics, Katholieke University Leuven (KU Leuven), 3000 Leuven, Belgium; 5 10x Genomics, Inc., Pleasanton, CA 94566 USA; 6 Department of Ecology and Evolutionary Biology, UC Santa Cruz, Santa Cruz, CA 95064 USA; 7 Department of Animal Science, University of Nebraska – Lincoln, Lincoln, NE 68583-0908 USA; 8 Department of Population Health and Reproduction, University of California, Davis, CA 95616 USA; 9 Veterinary Genetics Laboratory, University of California, Davis, CA 95616 USA; 10 Department of Veterinary Population Medicine, University of Minnesota, St. Paul, MN 55108 USA; 11 UF Genetics Institute, Department of Animal Sciences, University of Florida, Gainesville, FL 32611 USA; 12 Gluck Equine Research Center, Department of Veterinary Science, University of Kentucky, Lexington, KY 40546 USA; 13 Centre for GeoGenetics, Natural History Museum of Denmark, 1350K Copenhagen, Denmark; 14 Laboratoire d’Anthropobiologie Moléculaire et d’Imagerie de Synthèse UMR 5288, Université de Toulouse, CNRS, Université Paul Sabatier, Toulouse, France; 15 Baker Institute for Animal Health, College of Veterinary Medicine, Cornell University, Ithaca, NY 14853 USA; 16 Present Address: Medical and Molecular Genetics, Oregon Health and Science University, Portland, OR 97239 USA

**Keywords:** Genome informatics, Agriculture, Genomics, Next-generation sequencing

## Abstract

Recent advances in genomic sequencing technology and computational assembly methods have allowed scientists to improve reference genome assemblies in terms of contiguity and composition. EquCab2, a reference genome for the domestic horse, was released in 2007. Although of equal or better quality compared to other first-generation Sanger assemblies, it had many of the shortcomings common to them. In 2014, the equine genomics research community began a project to improve the reference sequence for the horse, building upon the solid foundation of EquCab2 and incorporating new short-read data, long-read data, and proximity ligation data. Here, we present EquCab3. The count of non-N bases in the incorporated chromosomes is improved from 2.33 Gb in EquCab2 to 2.41 Gb in EquCab3. Contiguity has also been improved nearly 40-fold with a contig N50 of 4.5 Mb and scaffold contiguity enhanced to where all but one of the 32 chromosomes is comprised of a single scaffold.

## Abstract (web summary)

Theodore Kalbfleisch et al. present an improved genome assembly for the domestic horse by combining short- and long-read data, as well as proximity ligation data. They improve contiguity of the assembly by 40-fold, with a 10-fold reduction in gaps.

## Introduction

The domestic horse *Equus caballus* is a culturally, economically, and historically important domesticated animal. Since horses were domesticated ~5 kya in central Asia^1^, humans have used them extensively for agriculture, transportation, military conflict, and sport. Horses have been selectively bred for speed, strength, endurance, size, appearance traits, and temperament.

EquCab2, a reference genome assembly of the domestic horse, was released in 2007^2^. This assembly was generated using the best genomic sequencing and assembly technologies available at the time, namely: Sanger sequencing, bacterial artificial chromosome (BAC) end pairs, radiation hybrid mapping, and fluorescence in situ hybridization (FISH) mapping. Since then, many researchers have used this reference genome to study the genetics of various traits in horses^3–9^, as well as their health^10–13^ and evolution^14–17^. However, EquCab2 contains numerous gaps in scaffolds as well as sequences unassigned to chromosomes, and genomic DNA resequencing^18^ and gene annotation^19^ studies have found inconsistencies in this genome. Therefore, new genomic technologies present an opportunity to improve the equine reference genome.

We present here a new reference assembly for the domestic horse, EquCab3. This assembly benefited from rapidly evolving high-throughput sequencing technologies and new algorithms used to assemble data from these platforms. Specifically, this project began from the solid foundation of 6.8-fold coverage Sanger sequence data^2^, as well as a radiation hybrid map and FISH data^20^. These data were augmented with 45-fold coverage Illumina short-read data that improved the characterization and accuracy of unique regions of the genome, increasing the contig N50 values 10-fold. Two different proximity ligation library preparation protocols made it possible to order these contigs and generate chromosome length scaffolds. In EquCab3, only chr6, with two scaffolds, is comprised of more than one scaffold. Finally, ~16× PacBio long reads made it possible to close many of the gaps between the ordered contigs, thereby improving the contig N50 values 4-fold again. The resulting assembly is enhanced not only in contiguity, but also in composition. This new version of the reference sequence for the domestic horse reduces the number of gaps 10-fold and increases the number of assembled bases by 3% in the incorporated chromosomes over EquCab2.

## Results

### A new reference assembly of the domestic horse genome

We generated a new reference assembly of the domestic horse using Sanger, short-read, and long-read sequencing data as well as physical chromosome maps. This new reference is derived from the same female Thoroughbred horse, Twilight, that produced EquCab2, and includes her mitochondrial genome.

We used both previously published data and newly generated data to generate this reference assembly. The previously published datasets are comprised of the data used to construct EquCab2: Sanger sequencing data, BAC-end pairs^2^, and a physical map containing radiation hybrid and FISH markers^20^. For this assembly, we generated shotgun Illumina short reads, Chicago and Hi-C proximity ligation libraries, PacBio long reads, and 10× Chromium linked reads. As there is no existing software or method for creating an assembly from this combination of data types, we developed a custom pipeline to leverage the strengths of each of these datasets.

First, we used the high coverage (45×) and accuracy of Illumina short reads to generate so-called super reads with MaSuRCA^21^. We assembled these super reads together with the long and accurate but lower coverage (6.8×) Sanger reads to create an initial assembly with Celera Assembler^22^. We scaffolded this initial assembly with the long insert-size Chicago and Hi-C proximity ligation libraries using the HiRise scaffolder^23^. To identify and correct misassemblies, we mapped all physical markers and sequence data, including BAC-end sequences, to the resulting scaffolds. We filled gaps in the corrected scaffolds with PacBio reads, which are longer than Sanger reads, using PBJelly. We phased the genome using 10× Chromium linked reads and the longranger^24^ pipeline. We aligned the high-identity and coverage Illumina short reads to the genome and used these alignments to correct errors. Finally, we used the physical map to assign scaffolds to chromosomes. The resulting assembly, EquCab3, is an improvement over EquCab2 in terms of contiguity, completeness, read mapability, and agreement with the physical map.

### Improved contiguity

EquCab3 has improved N50 values for both contigs and scaffolds over those reported for EquCab2: for the contigs, an N50 value of 4.5 Mb vs. 112 kb, and for scaffolds, 86 vs. 46 Mb (Table 1). At each phase of the assembly process (described in Methods section), there was an improvement in either the contig or scaffold N50 over the values achieved in EquCab2. The one exception is the scaffold N50 of the Sanger + MaSuRCa super reads. Our scaffold N50 is 6.6 Mb, less than the final value of 46 Mb reported in Wade et al.^2^ The EquCab2 value incorporated additional long-range data such as BAC-end reads from a library derived from Twilight’s half-brother Bravo, as well as radiation hybrid map data. With all PacBio and proximity ligation data from Twilight included, the contig N50 is increased 40-fold, and the scaffold N50 is increased from a chromosome arm-limited 46 Mb to a chromosome length-limited 86 Mb. Further, the total number of gaps in the ordered chromosomes is decreased more than 90%, from 42,304 gaps comprising 55 Mb (2.2% of the genome) in EquCab2 to 3771 gaps comprising 9 Mb (0.34% of the genome) in EquCab3.

**Table 1:** Resulting contig and scaffold N50s are presented here for each major step in the process of assembling EquCab3

Sequence composition	Contig N50	Scaffold N50
EquCab3		
Sanger + MaSuRCa super reads	1.2 Mb	6.6 Mb
Sanger + MaSuRCa super reads + Chicago + Hi-C	1.2 Mb	86 Mb
Sanger + MaSuRCa super reads + Chicago + Hi-C + PacBio	4.5 Mb	86 Mb
EquCab2		
Sanger Fosmid + BAC + Radiation Hybrid Map data	112 kb	46 Mb

For comparison, the contig and scaffold N50s for the final EquCab2 product are also shown. The final EquCab3 product (Sanger + MaSuRCa super reads + Chicago + Hi-C + PacBio) improved the contig N50 40-fold, and the Scaffold N50 was improved from a chromosome arm-limited 46 Mb to a chromosome length-limited 86 Mb

### Read mapping

**Fig. 1:**
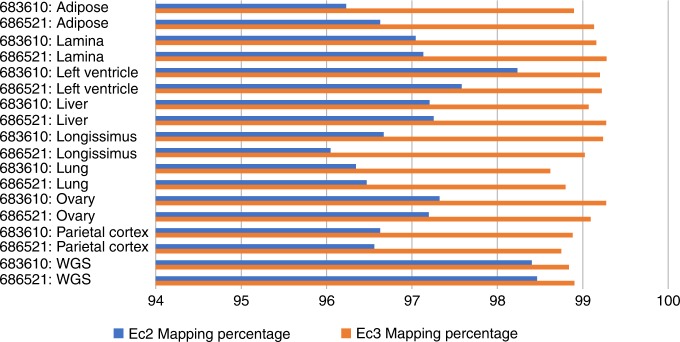
Percentages of RNA-seq reads from eight tissues from two horses (designated 683610 and 686521) and genomic reads mapping to EquCab2 vs. EquCab3. We used sequence data from the Functional Annotation of Animal Genomes (FAANG) project for this mapping. More RNA-seq reads map to EquCab3 than to EquCab2 for every tissue in both horses. The percentage of genomic reads (last two rows; WGS) mapping to EquCab3 is also larger than those mapping to EquCab2, but the difference is not as large

**Fig. 2:**
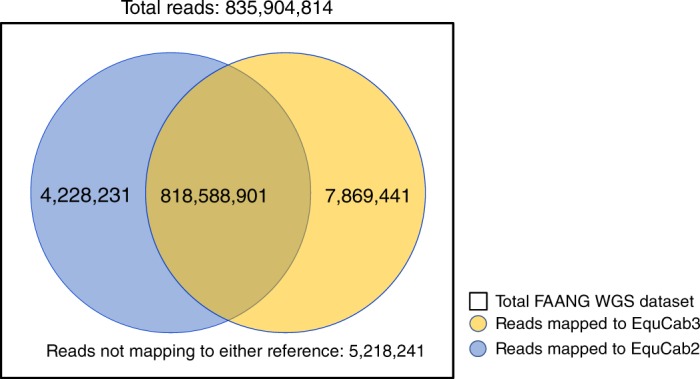
Number of reads from the Functional Annotation of Animal Genomes (FAANG) project WGS dataset mapping to EquCab2 and EquCab3. Significantly more reads map only to EquCab3 than only to EquCab2 (one-tailed two-sample binomial test *p* < 2.2 × 10^–16^)

The equine genome community is participating in the Functional Annotation of Animal Genomes (FAANG) project. The initial phase of this project has produced RNA-sequencing (RNA-seq) and whole-genome shotgun (WGS) sequence data from two Thoroughbred mares that are not the subject of the reference assembly. Data from both horses have been mapped to both EquCab2 and EquCab3^25^. The first phase of the equine FAANG effort was comprised of RNA-seq data  from eight tissues. As shown in Fig. 1, for RNA-seq, unique mappings of the reads are increased by an average of 2.15% over EquCab2, and WGS paired reads improved by 0.44%. All mapping count details are available in Supplementary Data 1. The count of reads mapping in a proper pair, that is, with both ends mapping with correct orientation, increased from a value of 811,622,501 to 814,804,213, an increase of 0.38% of the total read count. In addition, more reads in total (i.e., not limited to proper pairs) mapped to EquCab3 than EquCab2, as shown in Fig. 2.

**Fig. 3:**
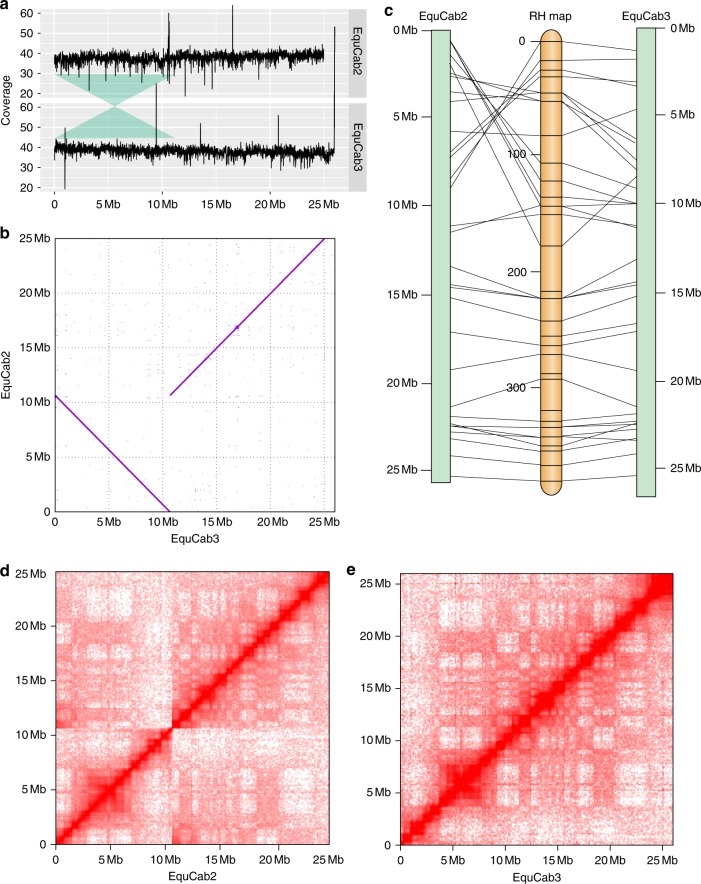
A comparison of equine chromosome 31 between EquCab2 and EquCab3. **a** Average coverage per 10 kb window across chr31 in EquCab2 and EquCab3, with a large inversion between them highlighted. EquCab3 has fewer coverage drops and more total sequence than EquCab2. **b** An alignment of chr31 in EquCab2 and EquCab3 shows a large inversion between the two reference genomes. The radiation hybrid (RH) map (**c**) and Hi-C contact heat maps for EquCab2 (**d**) and EquCab3 (**e**) indicate that this discrepancy is the result of a misassembly in EquCab2

This increase in read mapping is a function of several ways in which EquCab3 is an improvement over EquCab2. EquCab3 is more accurate due to the high-coverage high-identity Illumina data used both in the initial assembly and polishing steps.  EquCab3 also contains fewer gaps than EquCab2 due to the long-read gap-filling step, resulting in fewer dips in alignment coverage, shown in Fig. 3a. In addition, EquCab3 has more sequence assigned to chromosomes, giving reads more total sequence to map to, also demonstrated by Fig. 3a from the length increase in chr31 from EquCab2 to EquCab3. Finally, EquCab3 improves the characterization of GC-rich regions.

The total GC content of EquCab3 is roughly equivalent to that of EquCab2 (both near 41.6%). However, the guanine-cytosine (GC) fraction of the WGS reads for the two FAANG horses that mapped to EquCab3, but failed to map to EquCab2 is 48.9%. The GC content for the entire WGS dataset is 41.8%. This demonstrates an improvement in the characterization of GC-rich regions of the equine genome and is largely attributable to the PCR-free library preparations now in common use.

We also assessed the quality of EquCab3 by aligning ancient DNA (aDNA) reads to it. EquCab2 has been used in many studies as a reference for DNA recovered from paleontological samples, giving insight into the evolution and domestication of horses^14–17^. We compared mapping statistics between EquCab3 and EquCab2 for 13 previously sequenced ancient horses^17^ (Supplementary Data 2). A paired Wilcoxon's test showed a significant improvement in mapping (*p* = 0.0017), with all 13 samples having more reads mapped to EquCab3 than to EquCab2.

### Agreement with existing radiation hybrid map

We used a radiation hybrid map of the horse genome to assign scaffolds to chromosomes^20^. EquCab3 agrees with the radiation hybrid map more often than EquCab2. Of the 4103 markers on the physical map, 2982 map to EquCab2, while 3039 map to EquCab3. In addition, EquCab2 contains 391 marker pairs that are oriented differently on the assembly than on the map, whereas EquCab3 contains 395, despite the 57 additional markers mapping to EquCab3. This improvement can be attributed to the lower rate of misassemblies from the use of proximity ligation data for scaffolding. An example of a misassembly in EquCab2 corrected in EquCab3 is shown in Fig. 3b–e.

Of the 395 misoriented marker pairs on EquCab3, 352 are oriented the same way on both EquCab2 and EquCab3, but differently on the map. Twenty-one of the remaining 39 marker pairs that disagree with EquCab2 agree with EquCab3, while 18 do not have both pairs mapping to EquCab3. Given the multiple, orthogonal data types and differing assembly strategies used to construct EquCab2 and EquCab3, we suggest that some or all of these 352 marker pairs are oriented correctly in both assemblies, but incorrectly on the radiation hybrid map. Of the remaining 43 marker pairs that are misoriented on EquCab3 but not on EquCab2, 36 of these pairs do not have both markers mapping to EquCab2, leaving only seven marker pairs agreeing with EquCab2 but not EquCab3. Given that the radiation hybrid map was used to guide the assembly of EquCab2, we find this level of disagreement acceptable.

### Protein set completeness and comparative annotation

We used two methods to evaluate the completeness of our genome: universal ortholog analysis and comparative annotation. For universal ortholog analysis, we used BUSCO^26^ and the mammalian universal ortholog set. Out of 4104 mammalian universal orthologs, BUSCO found 4092 (99.7%) as complete orthologs in EquCab3 with 5 fragmented and 7 missing, compared to 4064 (99.0%) complete orthologs in EquCab2 with 27 fragmented and 13 missing. EquCab3’s higher BUSCO score indicates that it is more complete than EquCab2.

**Fig. 4:**
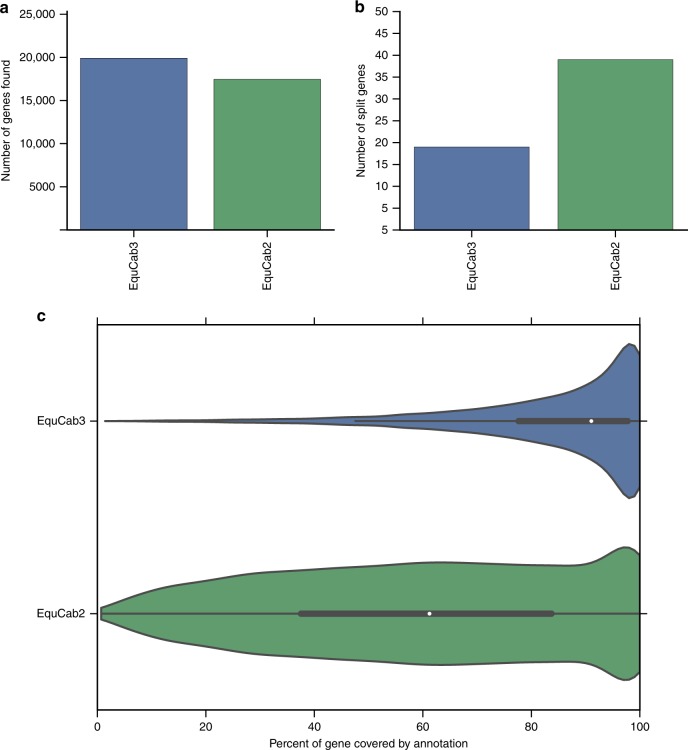
Annotation of EquCab2 and EquCab3 with the Comparative Annotation Toolkit shows substantial improvement in EquCab3. **a** More genes found in related species were annotated in EquCab3 than in EquCab2. **b** Fewer genes were split between contigs in EquCab3 than in EquCab2. **c** The gene coverage distribution is better in EquCab3 than in EquCab2

Comparative Annotation Toolkit (CAT) is a software pipeline that leverages whole-genome alignments, existing annotations, and comparative gene prediction tools to simultaneously annotate multiple genomes, defining orthologous relationships and discovering gene family expansion and contraction^27^. CAT also diagnoses assembly quality by investigating the rate of gene model-breaking indels seen in transcript projections from a reference, as well as looking at the rate of transcript projections that map in a disjointed fashion. We performed comparative annotation of EquCab2 and EquCab3 using the genomes of pig, cow, white rhinoceros, elephant, and human. Comparative annotation of EquCab3 and EquCab2 found more orthologs of genes in the other genomes in EquCab3 (Fig. 4a), fewer predicted genes split between contigs in EquCab3 (Fig. 4b), and a better distribution of gene coverage in EquCab3 (Fig. 4c). These results indicate that EquCab3 is a more complete and contiguous assembly than EquCab2.

### Phasing

Most published assemblies of diploid organisms are pseudo-haploidizations produced by arbitrarily choosing between the two alleles at each heterozygous site in the genome. The 10× Chromium platform is useful for haplotype phasing, as each set of linked reads it produces comes from the same haplotype. We took advantage of this by using 10× reads and the longranger^24^ pipeline to phase Twilight’s variants in EquCab3. For each phase block inferred by longranger, rather than arbitrarily choosing which haplotype to include in the final assembly, we chose the allele which is most common among four Thoroughbreds, the two FAANG horses, and data from two other Thoroughbreds from an earlier study by Sarkar et al.^12^ This makes the reference pseudo-haploidization more similar to the population average and thus more likely to contain the ancestral allele at each heterozygous site in Twilight’s genome. For analyses which would be adversely affected by this ancestral reference bias, we provide the phased 10× variant calls as supplemental data.

## Discussion

This new genome represents an improvement for the horse reference in terms of both composition and contiguity. It is also more consistent with the existing radiation hybrid map and FISH data for the horse than was EquCab2. Going forward, the lens through which this reference will be viewed will be as an alignment target for the vast amount of high-throughput sequence data that will continue to be generated for the horse and other related species. The assembly process described here was guided and informed by data that included not only high-quality short reads, but also long reads and proximity ligation data. All equine data produced by any of these technologies should be well served going forward. Illumina short reads, currently the most common data types for genetic and genomic studies, for two Thoroughbreds not related to Twilight or each other have been demonstrated to map to the new reference at an average rate of 99.06% for RNA-seq and 98.87% for WGS libraries, an increase of 2.15% and 0.44%, respectively. In a comparative genomics analysis, more gene orthologs were found, and for those that were found, the coverage of the homologous transcript sequence was more complete. The new long-range sequence data not only improved the contiguity of the genome, but allowed us to phase the genomic data for Twilight. Finally, the regions added for the genome were higher in GC content, which will enable a better characterization of both genetic variation and epigenetic status in GC-rich regulatory regions for the horse.

This represents a culmination of a project conceived and begun in 2014 with the support of the equine genomics community. Although it will certainly not be the last reference genome for the domestic horse produced for public annotation, it should foster genetic and genomic discoveries for years to come.

## Methods

### Sequence data generation

*Sample collection and DNA extraction*: A single reference horse was used for this study. Her care and collection of the blood sample for genomic DNA isolation are covered under the Cornell University Institutional Animal Care and Use Committee (IACUC) Protocol 1986–0216, which is approved from 21 January 2016 through 21 January 2019. Venous jugular whole-blood samples were collected from Twilight into evacuated tubes containing heparin. High molecular weight DNA was extracted from whole blood using the Qiagen Mag-Attract HMW DNA Kit (Qiagen, Valencia, CA, USA) following the manufacturer’s protocol. After isolation, DNA size and quality were determined using a Femto Pulse Automated Pulsed-Field Instrument (Advanced Analytical, Ankeny, IA, USA) at the Cornell University’s DNA Core Lab.

*Sanger data*: The sequence data set comprised of 30,111,484 Sanger reads for the Thoroughbred mare Twilight, produced for and used to build EquCab2^2^, were downloaded from the NCBI Trace Archive using the perl script query_tracedb downloaded from the NCBI website with the query string query page_size 30,000 page_number 223 binary SPECIES_CODE = EQUUS CABALLUS and CENTER_NAME = BI and CENTER_PROJECT = G836. In compliance with Trace Archive rules, 1004 individual shell scripts were executed to download the maximum 30,000 records per search request. This is described in more detail in Rebolledo-Mendez et al^18^.

*Illumina PE HiSeq and MiSeq*: Construction of a PCR-free shotgun genomic library and sequencing on MiSeq and HiSeq2500 instruments were carried out at the Roy J. Carver Biotechnology Center, University of Illinois at Urbana-Champaign (UIUC).

A shotgun genomic DNA library with an insert size of 500 bp (range 300–650 bp) was constructed from 2 µg of Twilight’s genomic DNA after sonication with a Covaris M220 (Covaris, MA, USA) with the Hyper Library Preparation Kit from Kapa Biosystems (Roche) with no PCR amplification. The DNA library with adaptors was loaded onto a 2% agarose gel and fragments 450 to 550 bp in length were cut from the gel and recovered with the QIAquick Gel Extraction Kit (Qiagen, CA, USA). The size-selected library was quantified with Qubit (ThermoFisher) and run on an Agilent bioanalyzer DNA high-sensitivity chip (Agilent, Santa Clara, CA, USA) to confirm the presence of DNA fragments of the expected size range. It was further quantitated by qPCR on a Bio-Rad CFX Connect Real-Time System (Bio-Rad Laboratories, Inc., Hercules, CA, USA) prior to sequencing for maximization of the number of clusters in the sequencing flowcell.

The PCR-free shotgun library was first sequenced on a MiSeq with v3 reagents to generate paired reads 300 nt in length. The fragment sizes were confirmed by measuring the distribution of insert sizes in the mapped MiSeq dataset. The mode of the distribution was 504 bp with an average insert size of 544.18 bp. The library was subsequently sequenced on two lanes of the HiSeq2500 for 151 cycles from each end using a TruSeq Rapid SBS Sequencing Kit1 v1. The fastq read files were generated with the bcl2fastq v1.8.4 Conversion Software (Illumina, San Diego, CA, USA).

*PacBio*: Ten micrograms of high molecular weight genomic DNA from Twilight was sheared with gTUBES (Covaris) in an Eppendorf^®^ 5424 centrifuge at 4800 RPM for 2 ×60 s. A single PacBio library was prepared from this following PacBio’s protocol P/N 100-286-000-07 (20 kb Template Preparation Using BluePippin™ Size-Selection System) with PacBio DNA Template Prep Kit 1.0. For the size selection, the sample was run on a 0.75% BluePippin cassette (ref: PAC20KB) using the pre-defined “0.75% DF Marker S1 high-pass 6–10 kb vs3” program and a cut-off of 10–50 kb. The library was sequenced on 88 SMRT cells on a PacBio RSII using DNA/Polymerase Binding Kit P6 and DNA Sequencing Kit 4.0 (v2) sequencing reagents, magbead loading, and stage start. All SMRT cells were run through PacBio’s SMRT Portal v2.3.0 pipeline RS_subreads.1 with default settings except for minimum subread and polymerase read lengths of 1 kb. In addition, reads of insert were generated using the RS_ReadsOfInsert.1 pipeline with a minimum insert read length set to 1 kb. Reads of insert had a mean of four passes and length of 11,785 bp.

 From the total initial read count of 5,934,426, we were able to create circular consensus (.ccs) reads totaling 371,943 reads. The remainder of the reads were used to generate an error corrected subreads file using canu^28^ (version 1.0) consisting of 5,562,483 reads. These two datasets were used in the PBJelly runs described below.

*CHiCago library*: We generated a CHiCago library as previously described^23^ using blood from Twilight.

*Hi-C library*: We generated a Hi-C library with primary fibroblasts from Twilight using a Hi-C protocol modified such that the chromatin immobilization took place on magnetic beads. We crosslinked the fibroblasts in formaldehyde, and lysed, washed, and resuspended as described by Lieberman-Aiden et al.^29^ We then immobilized the chromatin on SPRI beads as described by Deng et al.^30^ We restriction digested the DNA with *Dpn*II, labeled ends with biotinylated dCTP, ligated ends, and reversed crosslinks. The sample was prepared for sequencing using the NEB Ultra Library Preparation Kit according to the manufacturer’s instructions, with one exception: prior to indexing PCR, the sample was enriched by pulldown on 30 µL Invitrogen C1 streptavidin beads, and then washed to remove non-biotinylated DNA fragments.

*10× Genomics library*: Twilight’s genomic DNA was size selected for fragments >40 kb on a BluePippin instrument (Sage Sciences, Beverly, MA, USA) and Illumina sequencing libraries were constructed using the 10× Genomics Chromium Controller instrument with their Genome Reagents Kit v2 chemistry (10× Genomics, Pleasanton, CA, USA) according to the manufacturer’s recommendations. The resulting Illumina library was sequenced on a NextSeq500 using a High Output Kit v2 for a paired-end, 2 × 151 bp run (Illumina, San Diego CA, USA). The data were analyzed and assembled using the 10x Genomics Supernova version 1.1.5 pipelines.

### Assembly generation

*MaSuRCA*: We assembled all short reads from the Illumina genomic library into super reads using MaSuRCA^21^ version 3.1.3. The total length of sequences assembled into super reads was 4.7 Gb (approximately 2× coverage of the genome). These super read sequences had a contig N50 of 1734 nucleotides.

*Celera Assembler*: The Celera Assembler^22,31,32^, version 8.2 (downloaded from http://wgs-assembler.sourceforge.net/wiki/index.php?title = Main_Page), was used to create contigs and scaffolds using the super reads produced by MaSuRCA and the EquCab2 Sanger sequence data.

*HiRise*: We scaffolded the output of Celera Assembler using HiRise version 2.1.1 in a serial mode with default parameters, with the CHiCago and Hi-C libraries as input libraries^23^.

*Identifying misassemblies*: In order to identify misassemblies in the HiRise assembly relative to EquCab2, we aligned the HiRise output scaffolds to EquCab2 using nucmer with default parameters^33^. In every place where the alignment indicated a difference in order and orientation of scaffolds between the two assemblies, we used every available data type to resolve the discrepancy and determine which was correct. Our strategies included aligning BAC-end pairs from a half-brother of Twilight^2^ to the assemblies using bwa mem with default parameters^34^, assessing concordance with the physical map, looking for split genes predicted by the CAT^27^, aligning coding sequences of any genes in the region to the assemblies using gmap with default parameters^35^, and examining heatmaps of long-range read pairs mapping to the assembly generated by the HiRise and longranger pipelines^24^.

*PBJelly*: We filled gaps in the manually corrected HiRise scaffolds from the previous step using PacBio error-corrected subreads and circular consensus sequences as input to the PBJelly (version PBSuite_15.8.24) pipeline with the steps setup, mapping, support, extraction, assembly, and output, in that order.

*Assigning scaffolds to chromosomes*: We used a previously published radiation hybrid map^20^ to assign scaffolds to chromosomes. We aligned each physical marker’s short tandem sequence primers to the assembly using bwa fastmap^36^ and used only markers with both primers aligning uniquely and in the correct orientation. We then placed scaffolds on chromosomes based on the markers’ mapping locations.

*Mitochondrial assembly*: Illumina data were adapter trimmed using SeqPrep2^37^. A subset of 24 million randomly selected Illumina reads were created using seqtk sample^38^. The subsetted Illumina reads were used as input into an iterative assembler (mia version 1.0)^39^, using the horse mitochondrial sequence (NC_001640.1) as the reference with a slope of 175 and intercept of 100. Sanger data were used to determine the correct number of 8-mer repeats in the control region^40^. Sanger reads were trimmed using Figaro (version 1.05)^41^ and aligned to the initial mitochondrial assembly using bwasw (version 0.7.12)^42^. Alignments were manually inspected by eye using IGV (version 2.4)^43^. Sanger reads that aligned to the control region were extracted, visualized by eye, and compared to the initial mitochondrial assembly. One Sanger read spanned both sides of the control region 8-mer repeats and was used to update the number of 8-mer repeats in the mitochondrial assembly. The updated mitochondrial assembly sequence was used as the reference sequence for an assembly with mia, using 40 million randomly selected Illumina reads, with a slope of 180 and intercept of 100. The assembly was filtered such that sites not having at least 10-fold coverage and 90% consensus among bases were changed to N. The final assembly had an average coverage of 40×, and 6Ns. The new Twilight mitochondrial sequence has been deposited into GenBank (accession number MH586816).

### Quality control and assessment

*Read mapping*: Short-read sequence data generated in the initial phase of the equine FAANG project was mapped to both EquCab2 and EquCab3 for comparison of mapping fractions. These data can be found at the Sequence Read Archive. Both WGS sequence (40×) and RNA-seq (avg 20 M reads/tissue) datasets from eight tissue types for each of two animals were trimmed using TrimGalore (a wrapper for Cutadapt^44^). Full details including the project, biosample, and run accession numbers can be found in Supplementary Data 3. For WGS data, the program BWA^36^ (version 0.6.1) aln module was used to align the reads to the reference. BWA sampe was used to produce a SAM file. SAMtools^45^ (version 0.1.18) was used to convert from SAM to BAM format. Picard (version 1.65) FixMateInformation and MarkDuplicates modules were used, followed by GATK^46^ (version 1.5) RealignerTargetCreator, and IndelRealigner (validation_strictness set to LENIENT for each). For the RNA-seq data, the mapping program STAR^47^ (version 2.5.3a) was used with default parameters except for the following: --readFilesCommand zcat --outSAMtype BAM SortedByCoordinate --outBAMsortingThreadN 16 -outSAMunmapped Within.

*Polishing*: Since Twilight’s sequence data and EquCab3 were derived from the same animal, any homozygous differences between the PE data and the reference of which they are a component are likely errors. The differing bases were likely contributions from the sequence data generated on other platforms used for the assembly such as the Sanger or PacBio data.

The errors are either with the reference or with the miscalled/undersampled genotypes derived by the variant discovery software. To evaluate these positions, we performed variant discovery and genotyping with the UnifiedGenotyper using the Twilight PE data, the two FAANG thoroughbreds, and two additional thoroughbreds from Sarkar et al.^12^ whose data were downloaded from the Sequence Read Archive (see Data availability) and mapped as described above. The UnifiedGenotyper was used in discovery mode on the cohort. The resulting variant call format file was then parsed with custom java software^48^ looking for positions at which the Twilight data produced a homozygous genotype differing from the reference. The genotypes for the other animals were then queried at those positions. If the reference allele was detected in one of the other horses, the reference nucleotide at that position was not changed, with the idea that the second allele was either undersampled in the Illumina dataset or that a second allele was identified in the Sanger or PacBio sequence data.

*Removal of microbial contamination*: To build microbial sequence databases, all bacterial, viral, and fungal reference genomes were downloaded from RefSeq. For each of the three databases (bacteria, viruses, and fungi), the sequences were first masked with DustMasker^49^. Kraken v1.0^50^ was used to generate *k*-mers (*k* = 32) and to search the EquCab3 contigs for exact matches. Contigs with at least one exact 32-mer match were considered microbial contaminants and removed from the reference sequence. A total of 41 contigs were removed in this way.

*Removal of small contigs*: All scaffolds smaller than 3000 bases in length were removed from the assembly that was submitted for annotation. The contig and scaffold N50s for what was submitted were 4.73 and 87.2 Mb, respectively.

*Phasing with 10× data*: The data generated for Twilight on the 10× platform described above was mapped to the reference using the longranger (version 2.1.3) wgs module^24^. The phased variant file produced was then used to modify individual variant positions to conform to the haplotype whose allele was most common among the FAANG horses, and two other thoroughbreds described above.

*N50 calculation*: The PBJelly (version PBSuite_15.8.24) utility summarizeAssembly.py was used to calculate N50 values. The default setting of 25 was used for the minimum gap setting. This ignored any gaps sized <25 Ns.

*Universal ortholog analysis*: For universal ortholog analysis, we used BUSCO^26^ version 3.0.2 in protein mode with the lineage dataset mammalia_odb9 version 2016-02-13. For protein set inputs, we used the official NCBI protein sets for EquCab2.0 (accession GCF_000002305.2) and EquCab3.0 (accession GCF_002863925.1).

*Comparative annotation*: For this analysis, a progressiveCactus^51^ alignment of EquCab2 and EquCab3 was performed with pig (susScr3), cattle (bosTau8), white rhinoceros (cerSim1), elephant (loxAfr3), and human (hg38). The guide tree was (((Human:0.164501,((Pig:0.12,Cow:0.16908)1:0.02,(EquCab3:0.0001,EquCab2:0.0001):0.059397,White_rhinoceros:0.05)1:0.060727)1:0.032898)1:0.023664,Elephant:0.155646), putting EquCab2 and EquCab3 under the same node with a branch length of 0.0001. CAT^27^ was then run using the Ensembl V89 annotation of pig as the source transcript set. No RNA-seq data were provided, so no transcript cleanup steps or comparative gene predictions were performed. Split gene analysis was performed by looking at transcripts that had multiple projections after paralog resolution and that had multiple projections whose start and stop points were within 10 bp of each other in source transcript coordinates.

*Read filtering and counting*: Mapping locations that were not the primary mapping locations of reads were filtered with (getNotPrimaryAlignmentFlag() is false) within the mapped read (getReadUnmappedFlag() is false) count using htsjdk version 2.12.01^52^.

*Ancient DNA mapping*: We downloaded single-end Illumina reads produced by a previous study^17^ (Supplementary Data 2, NCBI Bioproject PRJEB19970). Adapters and PCR artifacts were trimmed using AdapterRemoval v2^53^. For normalization across samples, fastq files were downsampled to 6M reads using seqtk^38^. Low complexity sequences were removed using PRINSEQ^54^ following bwa mapping^36^ with parameters optimized for aDNA: *aln* algorithm, seed disable flag, and minimum mapping phred quality of 20.

### Code availability

The custom java software used in variant analysis can be found at https://github.com/kalbflei/EquCab3SingleNucleotideErrorCorrection.^48^

## Electronic supplementary material

Description of Supplementary Data

Supplementary Data 1

Supplementary Data 2

Supplementary Data 3

## Data Availability

The sequence read datasets generated during the current study are available in the NCBI SRA repository under accession SRP126689. The final assembly generated during the current study is available in the NCBI Genbank repository under accession GCA_002863925.1. The mitochondrial sequence has been deposited into GenBank (accession number MH586816). We also provide intermediate assemblies produced during the process, a de novo assembly based solely on the PacBio data, and phased variant calls from the 10× longranger pipeline in a CyVerse Data Commons repository at 10.7946/P20348^55^. Data from Sarkar et al.^12^ used in variant calling and genotyping are found in the Sequence Read Archive at BioSample SAMN03838869 and SAMN03838867, experiment accession numbers SRX1097022 and SRX1097495, respectively.
