# Author Correction: Improved reference genome for the domestic horse increases assembly contiguity and composition

**DOI:** 10.1038/s42003-019-0591-3

**Published:** 2019-09-11

**Authors:** Theodore S. Kalbfleisch, Edward S. Rice, Michael S. DePriest, Brian P. Walenz, Matthew S. Hestand, Joris R. Vermeesch, Brendan L. O’Connell, Ian T. Fiddes, Alisa O. Vershinina, Nedda F. Saremi, Jessica L. Petersen, Carrie J. Finno, Rebecca R. Bellone, Molly E. McCue, Samantha A. Brooks, Ernest Bailey, Ludovic Orlando, Richard E. Green, Donald C. Miller, Douglas F. Antczak, James N. MacLeod

**Affiliations:** 1 Department of Biochemistry and Molecular Genetics, School of Medicine, University of Louisville, Louisville, KY 40292 USA; 2 Department of Biomolecular Engineering, UC Santa Cruz, Santa Cruz, CA 95064 USA; 3 Genome Informatics Section, Computational and Statistical Genomics Branch, National Human Genome Research Institute, National Institutes of Health, Bethesda, MD 20892 USA; 4 Center for Human Genetics, Katholieke University Leuven (KU Leuven), 3000 Leuven, Belgium; 5 10x Genomics, Inc., Pleasanton, CA 94566 USA; 6 Department of Ecology and Evolutionary Biology, UC Santa Cruz, Santa Cruz, CA 95064 USA; 7 Department of Animal Science, University of Nebraska – Lincoln, Lincoln, NE 68583-0908 USA; 8 Department of Population Health and Reproduction, University of California, Davis, CA 95616 USA; 9 Veterinary Genetics Laboratory, University of California, Davis, CA 95616 USA; 10 Department of Veterinary Population Medicine, University of Minnesota, St. Paul, MN 55108 USA; 11 UF Genetics Institute, Department of Animal Sciences, University of Florida, Gainesville, FL 32611 USA; 12 Gluck Equine Research Center, Department of Veterinary Science, University of Kentucky, Lexington, KY 40546 USA; 13 Centre for GeoGenetics, Natural History Museum of Denmark, 1350K Copenhagen, Denmark; 14 Laboratoire d’Anthropobiologie Moléculaire et d’Imagerie de Synthèse UMR 5288, Université de Toulouse, CNRS, Université Paul Sabatier, Toulouse, France; 15 Baker Institute for Animal Health, College of Veterinary Medicine, Cornell University, Ithaca, NY 14853 USA; 16 Present Address: Medical and Molecular Genetics, Oregon Health and Science University, Portland, OR 97239 USA

**Keywords:** Genome informatics, Agriculture, Genomics, Next-generation sequencing

Correction to: *Communications Biology* 10.1038/s42003-018-0199-z, published online 16 November 2018.

In the original version of the article, the following funding information was incorrectly omitted from the Acknowledgements: “This project has received funding from the European Research Council (ERC) under the European Union’s Horizon 2020 research and innovation programme (grant agreement No. 681605 - PEGASUS).” The error has been corrected in the HTML and PDF versions of the article.

