# Supplementary material for: Improved reference genome for the domestic horse increases assembly contiguity and composition
Source: Commun Biol. 2018 Nov 16;1:197. doi: 10.1038/s42003-018-0199-z (PMC6240028; doi:10.1038/s42003-018-0199-z)
Supplement: Supplementary file 1 — Description of Supplementary Data [file 42003_2018_199_MOESM1_ESM.docx]

**Description of Additional Supplementary Files**

**File Name**: Supplementary Data 1

**Description**: Mapping Fractions for FAANG Whole Genome Sequence and RNA-Seq data, EquCab3 vs. EquCab2. Counts and fractions of RNA-Seq and WGS reads mapped to EquCab3 vs. EquCab2. These values are represented in the bar graph shown in Figure 1 in the manuscript. These data are described in more detail in Methods, Read Mapping.

**File Name**: Supplementary Data 2

**Description**: Mapping Statistics for Ancient DNA, EquCab3 vs. EquCab2. Comparison of mapping efficiency between EquCab2 (ecab2) and EquCab3 (ecab3) reference genomes. Each downloaded dataset was downsampled to ~6M reads. Raw mapping statistics represent the number of reads mapped to each genome after duplicate removal with samtools rmdup. The proportion of unique reads represent how many reads remain after duplicate removal. The last column represents a difference in the number of reads mapping to two genomes: raw number of deduplicated reads mapping to EquCab2 subtracted from number of reads mapping to EquCab3. These data are described in more detail in Methods, Read Mapping.

**File Name**: Supplementary Data 3

**Description**: NCBI Sequence Read Archive Accessions for FAANG Whole Genome Shotgun and RNA-Seq data used here. Sequence Read Archive accession numbers for RNA-Seq and WGS sequence data used in our validation step. The data is attributable to the Equine FAANG project (Finno, Bellone, and Petersen PIs). These data are described in Methods, Quality control and assessment.
